# Corrosion Behaviour of 316L Stainless Steel in CNTs–Water Nanofluid: Effect of Temperature

**DOI:** 10.3390/ma14010119

**Published:** 2020-12-30

**Authors:** Dana H. Abdeen, Muataz A. Atieh, Belabbes Merzougui

**Affiliations:** 1 Sustainable Development Division, College of Science and Engineering, Hamad Bin Khalifa University, Doha P.O. Box 34110, Qatar; bmerzougui@hbku.edu.qa; 2 Department of Chemical Engineering, College of Engineering, Qatar University, Doha P.O. Box 2713, Qatar; 3 College of Engineering, University of Sharjah, Sharjah P.O. Box 27272, UAE; dr.muatazali@gmail.com; 4 Desalination Research Group, Research Institute of Sciences and Engineering, University of Sharjah, Sharjah P.O. Box 27272, UAE; 5 Qatar Environment and Energy Research Institute (QEERI), Hamad Bin Khalifa University, Doha P.O. Box 5825, Qatar

**Keywords:** carbon nanotubes, gum Arabic, 316L stainless steel, potentiodynamic polarization, corrosion rate, polarization parameters, effect of temperature

## Abstract

The inhibition behavior of carbon nanotubes (CNTs) and Gum Arabic (GA) on the corrosion of 316L stainless steel in CNTs–water nanofluid under the effect of different temperatures was investigated by electrochemical methods and surface analysis techniques. Thereby, 316L stainless steel samples were exposed to CNTs–water nanofluid under temperatures of 22, 40, 60 and 80 °C. Two concentrations of the CNTs (0.1 and 1.0 wt.% CNTs) were homogenously dispersed in deionized water using the surfactant GA and tested using three corrosion tests conducted in series: open circuit test, polarization resistance test, and potentiodynamic scans. These tests were also conducted on the same steel but in solutions of GA-deionized water only. Tests revealed that corrosion increases with temperature and concentration of the CNTs–water nanofluids, having the highest corrosion rate of 32.66 milli-mpy (milli-mil per year) for the 1.0 wt.% CNT nanofluid at 80 °C. In addition, SEM observations showed pits formation around areas of accumulated CNTs that added extra roughness to the steel sample. The activation energy analysis and optical surface observations have revealed that CNTs can desorb at higher temperatures, which makes the surface more vulnerable to corrosion attack.

## 1. Introduction

Nanofluids are fluids with dispersed nanoparticles that have dimensions of less than 100 nm. Metallic and non-metallic fine particles can be suspended in base fluids such as water, oil and ethylene glycol, to have a fluid of enhanced physical and chemical properties [1]. The resulting nanofluids were found to have enhanced thermal conductivity, thermal diffusivity, convective heat transfer coefficients, and viscosity compared to their base fluids [2,3], which make them an efficient replacement for a heat transfer fluid (HTF) in many industrial applications [4].

The utilization of nanoparticles into HTFs provided several gains. Most importantly, the heat transfer enhancements were tremendous. Choi was the first to prove that a 160% enhancement of thermal conductivity for 1.0 vol.% CNTs in oil was achieved, which is unusual and more than one order of magnitude higher than the theoretical models [5,6]. Furthermore, the implementation of nanoparticles in the HTFs conquered some of the troublesome problems related to the use of micro-sized particles such as increased pressure drop, poor stability, clogging pipelines, higher pumping power and erosion problems [7,8,9]. Using nanofluids in a heat transfer system increases its efficiency, which decreases the fuel consumption and reduces the required area of the system [10,11]. This has achieved economic and environmental savings related to energy and reductions in the emissions of greenhouse gases [12,13]. These new innovative fluids have unique characteristics that caused revolutionary enhancements in various applications.

Such enhancement in physical and chemical properties of the nanofluid is due to the presence of the nanoparticles. Adding small amount of metallic or non-metallic nanoparticles to a HTF decreases its thermal resistance hence increases its thermal conductivity. The percentage of thermal conductivity enhancement depends on many factors such as the size, shape and loading of the particles [14,15], the preparation method of the fluid and of the particles [16], thermal conductivity of the base fluid [17], presence of additives, flow conditions [18], temperature [19], and pH of the fluid [15,20], etc. Enhancement in thermal conductivity can reach up to 40% with the addition of 0.3 vol.% of Cu to ethylene glycol [21]. Furthermore, multi-walled carbon nanotubes (MWCNTs) suspended in water with 0.25 wt.% Gum Arabic (GA) at temperature of 30 °C attained a maximum thermal conductivity enhancement of 18% and 37% using 0.1 and 0.5 wt.% CNT concentrations, respectively [18]. A 0.1 wt.% CNT nanofluid of 1:1 CNT:GA ratio resulted in 12.1% increase in the thermal conductivity of the CNTs–water nanofluid [22].

With the introduction of nanofluids to heat transfer systems, an enhancement in heat transport properties was previously investigated. However, it is important to maintain safety consideration with the utilization of nanofluids in regard to corrosion performance as most base. HTFs originally has corrosion problems without the addition of such particulates [23]. The presence of nanoparticles in the base fluid alters its thermo-physical properties, hence the reactivity of nanofluids with the surroundings should be considered.

Corrosion is a vital aspect that should be considered in any engineering system to maintain process performance and prevent systems failure [24]. In the US, corrosion cost had a share of 6% of its gross domestic products (GDP) [25], while globally it accounts for 3.4% of the global GDP [26]. Not only do corrosion damages affect the economic aspects of a project, they can also cause serious safety influences in the form of explosions and fires, release of toxic products, human injuries, etc. [27]. The corrosion rate is affected by several factors related to the surroundings such as temperature and pH, or related to the constituents and the structure of the material exposed to these conditions [28]. Identifying corrosion in its early stages is important to mitigate and control corrosion once it happens. Utilizing nanofluids around the metals creates different surroundings that should be considered whether it would damage and deteriorate the exposed surfaces.

Previous work done in the area of corrosion of nanofluids is very few. Erosion effect of oxides nanoparticles was examined using the HETNA (hydraulic experiments on thermo-mechanics of nanofluids) apparatus, and it was revealed that the occurred weight loss of the metallic samples was due to chemical corrosion rather than mechanical erosion [29,30]. With the immersion test and while the temperature was increasing from 27 to 92 °C, Rashmi et al. reported on lower corrosion rates when exposed to CNTs nanofluids, having the aluminium samples of highest corrosion rates with respect to stainless steel and copper samples [31]. Srinnivas and Moorthy noted that the addition of CNTs to an automotive coolant did not alter the corrosion performance of the immersed metallic samples at 88 °C [32]. Furthermore, the addition of CNTs to a solution with SDS or SDBS surfactants at room temperature did not considerably change the corrosion rate of carbon steel samples, while the functionalized CNTs caused a small reduction in the corrosion rate [33]. Most of the previously-mentioned work has not examined the effect of CNTs nanofluids on the corrosion of the samples, and were focusing on other factors such as heat transfer properties, surfactants or additives [31,32], agitation [34] or erosion effect [29,30]. Abdeen et al. studied the effect of different CNTs concentration on the corrosion of 316L stainless steel at room temperature. The addition of CNTs has decreased the corrosion rate, but higher CNTs concentration increased the corrosion rate due to unevenly and non-uniformly distributed CNTs over the surface of the samples [35]. The present work is a continuation of the last one mentioned. It is mainly focused on the corrosion effect of exposing the tested samples to different temperatures of the CNTs–water nanofluid that is dispersed in the base fluid using the surfactant Gum Arabic. Thus, this study aims to assess the influence of the CNTs’ presence in the tested solution on the steel surface when the steel is exposed to different temperatures of that nanofluid, and the nature of CNTs adsorption while being exposed to such environment.

Utilizing the CNT-water nanofluid as HTF must be considered from the corrosion aspect which has not been studied thoroughly. While exploiting the nanofluids into thermal applications, they are being exposed to higher temperatures which would influence the mechanical properties of the CNTs [36] and the inhibition efficiency of the GA surfactant [37,38], hence their response to adjacent surfaces will be affected. The material selected is 316L stainless steel as it is commonly used in thermal applications especially inside heat exchangers’ tubes. The current research investigates the effect of different temperatures of the CNTs–water nanofluid (22, 40, 60, and 80 °C) on the corrosion of the 316L stainless steel samples that are tested in 0.1 and 1.0 wt.% of the same nanofluids. Surface examinations of SEM and optical profiler observations in addition to the contact angle analysis were performed to further understand the corrosion performance.

## 2. Experimental Work

Nanofluid used in testing is CNTs–water, where CNTs are dispersed in deionized water using GA surfactant. Nanofluid was synthesized using two-step technique, where CNTs were first synthesized then dispersed in water. MWCNTs were purchased from Cheaptubes Company (Grafton, VT, USA). They were produced through catalytic chemical vapour deposition (CCVD) process and then treated using concentrated acid chemistry method to obtain a purity higher than 95%. Specifications of the MWCNTs are presented in Table 1.

Gum Arabic (GA) was used as a surfactant agent to ensure a homogenous dispersion of the hydrophobic CNTs in water. GA, purchased from Sigma-Aldrich, was used since it has proved to sustain high temperatures without foaming [39]. GA was added to deionized water with a weight ratio of 1:3 (CNTs: GA), then sonicated at room temperature for one hour in an ultrasonication probe (vibra-cell) from Sonics & Materials, Inc. (Newtown, CT, USA). No visual sedimentation was observed after one month of nanofluid synthesis. In addition to synthesized CNTs nanofluids, samples were tested in GA only solutions (no CNTs added) that were prepared by dissolving same amounts of GA in deionized water. Average pH values for CNT-water nanofluids and GA solutions was 5.6 and 5.3, respectively. Finally, tap water was also used as a test solution. It was analyzed in Gulf Laboratories CO. W.L.L and found to have the composition listed in Table 2 [40]. Stability, thermal conductivity, and viscosity of the nanofluids used in this research have been measured in the same lab and published in another work [22].

Samples used in testing are 316L stainless steel, with the composition shown in Table 3. An annealed cylindrical rod of 1/4” (6.35 mm) diameter X 2-1/2” long (63.50 mm), were purchased from Metal Samples (Munford, AL, USA), a division of Alabama Specialty Products, Inc. They were cut, glued with conductive adhesive to a copper wire, and then mounted in an epoxy resin leaving one side exposed as a test surface. The test surface was polished using an automatic grinder-polisher with up to 1200-grit paper.

A three consecutive corrosion tests were conducted for each sample; open circuit potential test (OCP), polarization resistance test, and potentiodynamic scan test. An Interface 1000 potentiostat and EuroCell kit from Gamry Instruments (Warminster, PA, USA) were used to obtain the corrosion parameters. The corrosion cells were immersed in a Fisher Scientific–Isotemp heater bath to maintain the required temperature during the test. The ASTM G59-97 and ASTM G5-14 standards were followed to perform polarization resistance test and potentiodynamic scan, respectively [41,42]. A saturated calomel electrode (SCE) was used as a reference electrode, and a graphite rod was used as a counter electrode. The counter electrode was placed in a nafion membrane to reduce the high resistance developed in the corrosion cell due to the testing in deionized water and presence of CNTs particles. Polished stainless steel samples were rinsed with ethanol then with deionized water before testing. They were immersed in test solution 10 h before testing to allow stabilization of the surface of the sample. The polarization resistance test was run at a scan rate of 0.6 V/hr (0.167 mV/s), and under potential range of 0.25 V above and below the open circuit potential that was obtained from OCP test. Potentiodynamic test was performed at the same scan rate, and the test was run from −0.25 V below open circuit potential to 1.5 V. After these corrosion testing, the samples were allowed to air-dry before further observations. Experimental examinations were conducted for the two CNTs concentrations of 0.1 and 1.0 wt.% CNT-water nanofluid and each nanofluid concentration was tested at temperatures of 22, 40, 60, and 80 °C.

## 3. Discussion of Results

### 3.1. Corrosion Potential (Ecorr)

**Figure 1:**
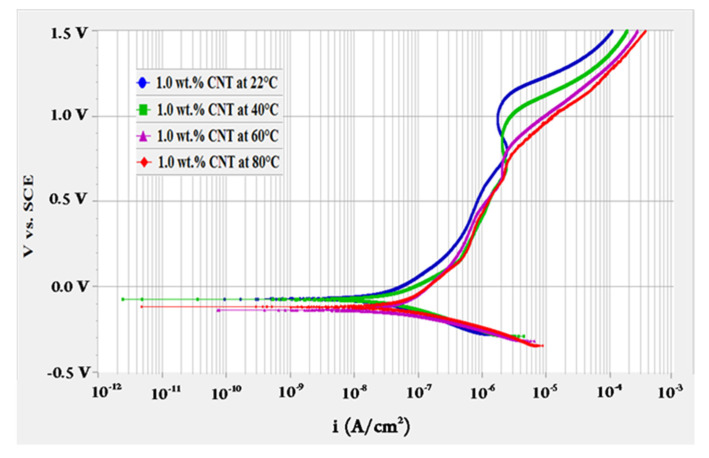
Potentiodynamic scan for 316L stainless steel tested in 0.1 wt.% CNTs + 0.3 wt.% GA in deionized water nanofluids at different temperatures.

**Figure 2:**
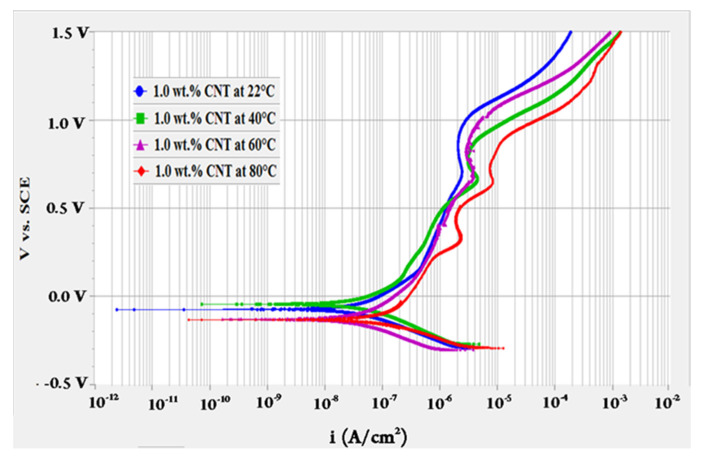
Potentiodynamic scan for 316L stainless steel tested in 1.0 wt.% CNTs + 3.0 wt.% GA in deionized water nanofluids at different temperatures.

To evaluate the effect of fluids’ temperature, OCP test was performed for 10 h followed by polarization resistance test then a potentiodynamic scan at different temperatures of 22, 40, 60 and 80 °C. The potentiodynamic scans of 0.1 and 1.0 wt.% CNTs–water nanofluids at different temperatures are shown in Figure 1 and Figure 2, respectively. These curves represent the change in the anodic and cathodic reactions that happen on the surface of the tested sample. They also give indications of the passive behaviour (oxides formation) on the surface while interacting with the surroundings. As revealed in Figure 1, the change of current density with the potential had almost similar behaviour for the 0.1 wt.% CNTs nanofluids. For the 1.0 wt.% CNTs nanofluids in Figure 2, a comparable behaviour evolved for all temperatures, whereas the highest temperature of 80 °C had several turnings. Turnings in the curves of both figures were due to the formation of different types of oxides at the surface of the metal where the potential increased with the time [43]. Curves followed passive-active-transpassive-active behaviour, although for the moderate temperatures (40 and 60 °C) of the 0.1 wt.% CNTs the transpassive regime did not completely appear. Both concentrations had a passivation current density for all temperatures in the range of 0.25–1.22 µA/cm^2^ with a passivation potential between 0.12–0.54 mV_SCE_. These values were obtained from the polarization curve where the current density had a change of less than one order of magnitude.

CNTs are carbon molecules arranged in rolled layers to form a cylindrical shape of few micrometers long. They were suspended in deionized water through GA surfactant, which was chosen because it can form more stable solutions especially at high temperatures [39]. GA is an organic inhibitor of complex structure [44] that can reduce corrosion attack by being physically or chemically adsorbed on the surface of the metal [44,45]. GA molecules can be adsorbed into the surfaces through their functional groups, namely hydroxyl (–OH) and carboxyl (–COOH) ones [46]. The inhibition potential of a polymeric compounds such as GA depends on their molecular structure and chemical composition, surrounding conditions of composition and temperature, in addition to the nature of the surface to be protected [47]. Temperature affects the adsorption mechanism of GA, as it was physically adsorbed on a mild steel in 0.1 M H_2_SO_4_ [37], while it was chemically adsorbed in the same media but on another mild steel of different composition [45]. Furthermore, GA concentration influences its inhibition efficiency, as GA protection for carbon steel increased in HCl solution with GA concentration up to 4 g/L, after that concentration the inhibition did not change significantly [48]. Acacia seyal gum decreased the corrosion rate of steel in potable water from 2.911 to 0.046 mpy for uninhibited and inhibited water, respectively. Steel protection increased with increasing gum concentration and remained constant after adding 600 ppm of the gum [49].

In the current work, the same corrosion testing was conducted for GA only solutions of the same amount of GA presents in the 0.1 and 1.0 wt.% CNTs nanofluids. Corrosion potential values for tested CNTs nanofluids at different temperatures along with their difference with respect to GA-deionized water solutions of corresponding concentrations are presented in Table 4.

The values of E_corr_ obtained with the corrosion experimentation in CNTs nanofluids and in GA only solutions are compared. E_corr_ values are obtained from the extrapolation of the Tafel lines when the electrode is polarized. Changes in E_corr_ with the addition of species determine the dominant reaction/s during the corrosion process. If the shift in E_corr_ due to the addition of certain species is less than 85 mV, then both reactions (cathodic and anodic) have been affected. However, if the difference is more than 85 mV, then the addition of the species has influenced one reaction only. The shift direction, either positive or negative, indicates that these species have retarded only anodic or only cathodic reaction rate, respectively [50].

The anodic reaction in the aerated neutral solution here is the dissolution of the metal ($Fe_{\left(s\right)}\;\to \;Fe^{+2}{}_{\left(aq\right)}+2\;e^{-}$), and the cathodic reactions are the water reduction ($H_{2}O+\frac{1}{2}\;O_{2}+2\;e^{-}\;\to \;2OH^{-}$) and hydrogen gas evolution ($2\;H^{+}{}_{\left(aq\right)}+2\;e^{-}\;\to \;H_{2}{}_{\left(g\right)})$. Since the displacement in E_corr_ of the CNTs nanofluids from their corresponding ones of GA solutions are less than 85 mV, then addition of CNTs at all temperature ranges has influenced both reactions. However, it can be noticed that there is a dominant effect for retarding the anodic reaction more than the cathodic reaction due to the positive displacement of E_corr_ for the CNTs nanofluids from the corresponding E_corr_ values for GA solutions. Hence, CNTs affected both metal dissolution reaction and hydrogen reduction reaction, and their influence was higher on the former one as they worked on reducing the corrosion rate by occupying anodic sites. Retarding the anodic reaction effect is applicable for both concentrations of the CNTs at the tested temperatures up to 80 °C.

Adsorption is the mechanism to be suggested for inhibition mechanism of CNTs and GA of the stainless steel surface. An inhibitor can reduce metal dissolution through (1) blocking effect that works by covering the surface and reducing available area for corrosion attack (2) surface energy effect, where the inhibitor works into changing the surface activation energy needed for redox reactions [51]. Hence, the amount of E_corr_ displacement for the steel tested in CNTs nanofluid with respect to that when the test solution has no CNTs can provide an insight as to whether the adsorption mechanism of CNTs/GA was more supported with surface energy or with the blocking effect. Ideally, no change in the E_corr_ with the presence of inhibitor points out the higher influence of blocking geometry compared to the surface energy effect [50,51]. In our case, the shift in E_corr_ with the addition of CNTs compared to GA-deionized water solutions was slightly towards more positive values at all temperatures. The largest shift in E_corr_ (ΔE_corr_) of 82 mV_SCE_ was noticed when the steel samples were tested in 0.1 wt.% CNTs nanofluids at 22 °C. Furthermore, for both CNT concentrations and at higher nanofluids temperatures of 60 and 80 °C, ΔE_corr_ was very small so that the blocking effect is substantially leading the inhibition process. Such a result signifies the higher effect of CNTs blocking geometry with a minor surface energy influence, especially at higher temperatures. Inhibition of CNTs through blocking effect was also obtained in the first part of this study, where the same 316L stainless steel was tested in CNTs–water nanofluids of different CNTs concentrations at 22 °C [35].

### 3.2. Polarization Parameters

Corrosion testing conducted on the 316L stainless steel at different temperatures provided various polarization parameters that are presented in Table 5. Data are obtained by selecting the inspected region to be ±50mV around the OCP value. These values are considered accurate to an acceptable level as the software is consistent in finding these values with minimal errors. Anodic and cathodic Tafel slopes (β_a_ and β_b_) represent the slope of the anodic and cathodic parts of the potentiodynamic curve, respectively. They were obtained through Gamry software with specifying the inspected potential range to be ±50 mV around the open circuit potential. β_a_ and β_b_ were changed with the addition of 0.1 and 1.0 wt.% CNTs at all temperature ranges indicating that the addition of CNTs and GA has affected both reactions. This is consistent with the fact that GA is a mixed type inhibitor, that is to say GA inhibits the corrosion by retarding both anodic and cathodic reactions [46,49,52]. In addition, the finding that the presence of 0.1 and 1.0 wt.% CNTs nanofluids influenced both reactions at temperatures up to 80 °C is in agreement with the results reported previously where a slight displacement of E_corr_ values was noted for CNTs nanofluids compared to GA-deionized water solutions.

**Figure 3:**
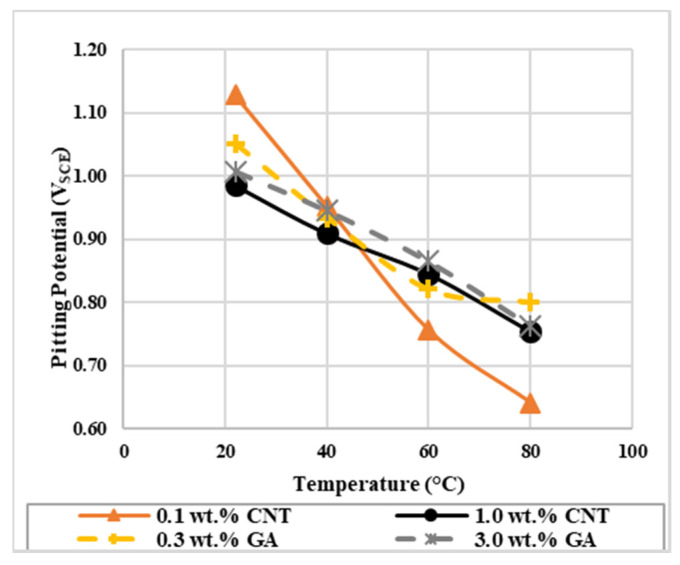
The effect of temperature on the pitting potential of 316L stainless steel exposed to CNTs–water nanofluids and GA-deionized water solutions of different concentrations.

Pitting potential (E_pit_) is another important polarization parameter obtained from the potentiodynamic test. E_pit_ is an indicator for the tendency of the material to corrode in a specific condition. It can identify pit nucleation and detect the effect of surface and surrounding conditions on pit initiation, hence E_pit_ can help in determining the acceptance or rejection of a specific material on the commercial scale [53,54,55,56]. E_pit_ is located at the potential where a significant increase in the current density is noticed in the potentiodynamic curve. The average values of E_pit_ are summarized in Table 5 and the comparison graph is shown in Figure 3. For both concentrations of CNTs nanofluids and at all temperature ranges, E_pit_ values for CNTs nanofluids were lower than the corresponding values of GA-deionized water solutions, except for 0.1 wt.% CNTs at 22 and 40 °C. For all tested solutions, E_pit_ values decreased when the experiment was performed at higher solution temperatures. At lower temperatures, the pitting attack started at lower potentials for the 1.0 wt.% CNTs nanofluid compared to the 0.1 wt.% nanofluid. While at higher temperatures, the pitting attack started earlier for the 0.1 wt.% nanofluid compared to the 1.0 wt.% nanofluid. This indicates that the susceptibility to pit initiation depend on both; the concentration and temperature of the nanofluid. Hence, a faster pit attack was observed at lower and higher temperatures for the 1.0 wt.% and 0.1 wt.% CNTs nanofluids, respectively. Since it was inferred from E_corr_ values for the nanofluids that blocking effect had stronger influence on the inhibition process, lower E_pit_ obtained might be due to decreased number of CNTs and/or GA species that blocked the surface from further dissolution. Also, a desorption of any of the attached species might have happened at higher temperatures and concentrations that caused the pitting to initiate at lower potentials [50].

Polarization resistance (R_p_) is a corrosion parameter that reveals the inhibition performance. It is inversely proportional to corrosion rate values and it was showed to decrease with increasing CNTs/GA concentrations and temperature. R_p_ values were mostly higher than the corresponding ones found in GA solutions when the steel was tested in CNTs nanofluids, indicating the presence of an extra resistance in the test solution. The highest R_p_ value of 5950 kohm was obtained for 0.1 wt.% CNTs nanofluid at 22 °C.

**Figure 4:**
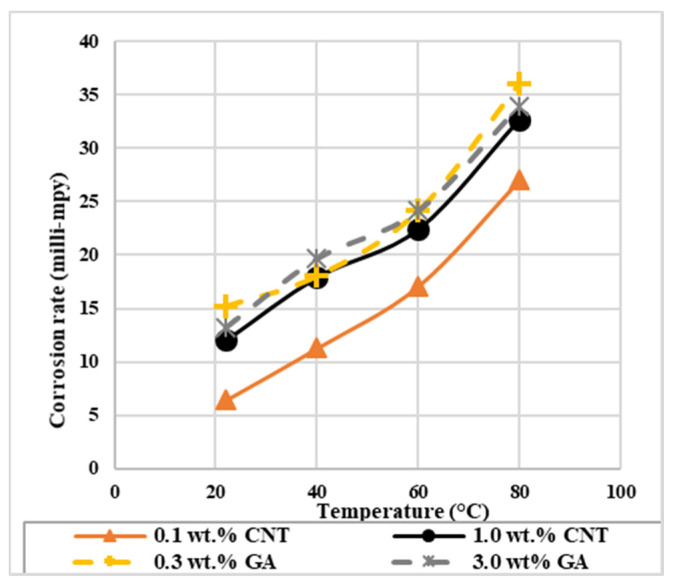
The effect of temperature on the corrosion rate of 316L stainless steel exposed to CNTs–water nanofluids and GA-deionized water solutions of different concentrations.

With the presence of CNTs on the surface of the steel, corrosion rate values in Table 5 was found to change with nanofluids concentration and temperature. In general, the corrosion rate of the steel tested in nanofluids at all temperatures are lower than their corresponding values for GA-deionized water solutions, as shown in Figure 4. This result implies that CNTs decreased the corrosion rate by being adsorbed on the surface of the metal and retarded its dissolution. The lowest corrosion rate of 6.43 milli-mpy (milli-mil per year) was obtained with 0.1 wt.% CNTs nanofluids at 22 °C, and the highest value of 32.66 milli-mpy was found with the 1.0 wt.% CNTs nanofluid at 80 °C. For each nanofluid concentration, higher temperatures decreased CNTs inhibition as it is revealed from the rise in the corrosion rate values with temperature. This might be due to the desorption of some CNTs from the surface. Furthermore, the increase in CNTs concentration from 0.1 to 1.0 wt.% at a specific temperature increased the corrosion rate. With the presence of CNTs and GA in the solution, both species provide a barrier and contribute in increasing the mass transfer resistance to attack the surface of the metal. However, the distribution of CNTs on the surface of the metals are not homogenous. At higher CNTs concentrations, they might accumulate randomly causing active anodic sites, which will promote corrosion [35].

Concerning the GA-deionized water solutions, the corrosion rate decreased with increasing temperature for both concentrations. GA inhibition can increase [45], decrease [37], or provide no change [48] with the temperature increase, and this is subject to the adsorption mechanism of GA, whether it is a physical or chemical type [46]. Increasing the corrosion rate of GA with temperature is an indication for a physically adsorbed GA on the surface of the 316L stainless steel [48,57,58]. Moreover, with increasing GA concentration from 0.3 to 3.0 wt.% in the deionized water, the corrosion rate remained almost the same at each corresponding temperature. GA inhibition may increase with concentration but after a certain limit, the corrosion rate stays constant. So, the surfactant did not work as corrosion inhibitor. The same was achieved with SDBS surfactant that was used to stabilize CNTs–water nanofluid [33]. This was also revealed for the inhibition of GA surfactant in potable water that has GA concentration up to 600 ppm, where after this concentration GA inhibition did not exhibit any change [49].

The inhibition performance of CNTs can be examined by comparing the corrosion rate of CNTs nanofluid with respect to the base solutions of no-CNTs solutions, which are here the GA-deionized water solutions. Table 5 shows the inhibition efficiency (IE%) values for each nanofluid that were calculated as the difference between the corrosion rate when the test was performed with and without CNTs, and with respect to the no CNTs solutions (GA deionized water). The highest inhibition percentage of 57.9% was obtained with CNTs nanofluid of 0.1 wt.% at 22 °C, and the lowest value was 3.5% for the 1.0 wt.% nanofluid at 80 °C. It was noted that the inhibition in 0.1 wt.% CNTs nanofluid was higher than the corresponding values in 1.0 wt.% nanofluid at each temperature. In addition, the inhibition of CNTs decreased with temperature for both CNTs loading, but this decrease was marginal and almost the same for the higher CNTs loading.

### 3.3. Activation Energy and Other Thermodynamic Properties

**Figure 5:**
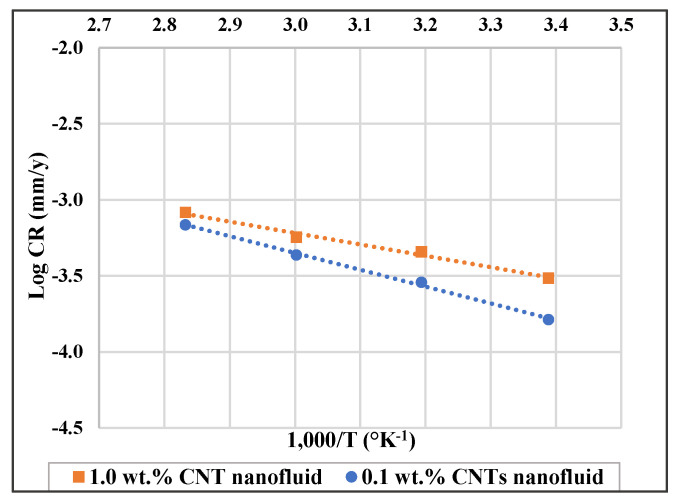
Log corrosion rate (log CR) versus (1000/T) for 316L stainless steel in 0.1 wt.% and in 1.0 wt.% CNTs–water nanofluids.

Corrosion of 316L stainless steel was studied in 0.1 and 1.0 wt.% CNTs–water nanofluid at temperature range of 22–80 °C. The corrosion rate of the steel was found to decrease with the addition of CNTs, however it increased with higher concentrations and temperatures. Higher temperature can influence the inhibition process as it might involve rupture, etching, and desorption of some adsorbed molecules. The inhibition percentage might increase or decrease as temperature increases depending on the adsorption strength of the adsorbed molecules, whether it is a physical electrostatic interaction or a chemical covalent bonding [59]. The mechanism type can be inferred from some thermodynamic properties such as the activation energy (E_a_), enthalpy of adsorption (ΔH_a_) and Gibbs free energy of adsorption (ΔG_ads_). With the logarithm of the corrosion rate (log CR) to the reciprocal of temperature (1/T) (Figure 5), a liner relation was obtained, which indicates that it follows Arrhenius equation as follows:

(1) $$\log CR=\log A-\frac{E_{a}}{2.303\;R\;T}$$

where *CR* is the corrosion rate, *A* is Arrehenius constant, *R* is the gas constant, and *T* is absolute temperature. The E_a_ values are obtained from the slope (−Ea2.303R) of the above equation. For low E_a_ values (E_a_ ranges between 0 and 40 kJ/mol), energy requirement for surface adsorption are small, and physical adsorption is usually taking place, as it is a rapid and an easily reversible process. For higher E_a_ values (E_a_ ranges between 40 and 800 kJ/mol), energy needs for adsorption are higher and chemical adsorption is occurring that involves strong forces [60]. As presented in Table 6, E_a_ values with the CNTs nanofluids are higher than the corresponding ones for GA-deionized water solutions of the same concentration. A higher activation energy means a higher energy barrier of the corrosion reactions to occur hence higher inhibition efficiency is expected to obtain [61,62]. The activation energy values for the stainless steel in 0.1 wt.% CNTs nanofluid and in 0.3 wt.% GA solution were 21.2 and 12.7 kJ/mol, respectively. This indicates that the addition of CNTs worked on retarding corrosion by being physically adsorbed on the surface and hence by forming a layer that is electrostatically attached to the surface of the metal [37].

**Figure 6:**
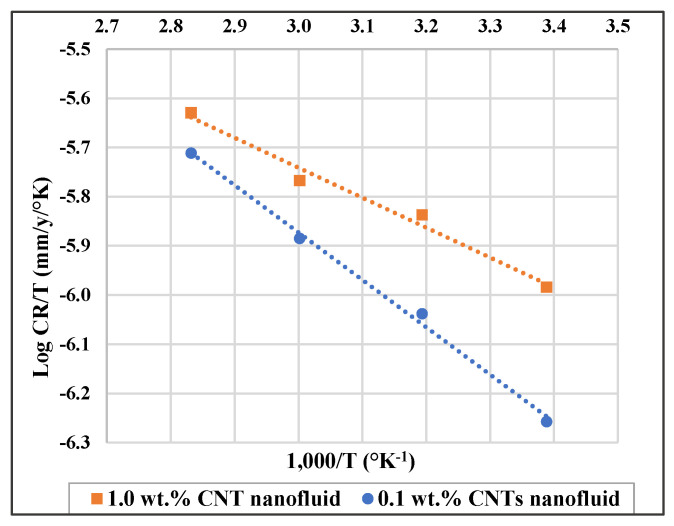
Log CR/T versus (1000/T) for 316L stainless steel in 0.1 wt.% and in 1.0 wt.% CNTs–water nanofluids.

Furthermore, enthalpy of adsorption (*ΔH_a_*) and entropy of adsorption (*ΔS_a_*) can be obtained by applying the below transition state equation [48,63,64,65]:

(2) $$\log\frac{CR}{T}=\left[log\left(\frac{R}{N\;h}\right)+\left(\frac{\Delta S_{a}}{2.303\;R}\right)\right]-\frac{\Delta H_{a}}{2.303\;R\;T}$$

where *N* is the Avogadro’s number, *h* is Planck’s constant, *R* is gas constant, and *T* is the absolute temperature. A plot of log (CR/T) against (1/T) is shown in Figure 6, where ΔS_a_ and ΔH_a_ can be found from the slope (−ΔHa2.303 R) and intercept (log(RN h)+(ΔSa2.303 R)), respectively. ΔS_a_ and ΔH_a_ values are shown in Table 6.

If the absolute value of ΔH_a_ is between 2.1–20.9 kJ/mol, then it is a physical adsorption process, while for higher values between 80–200 kJ/mol, the species are chemically adsorbed into the surface of the substrate [60]. Therefore, based on the ΔH_a_ obtained, our process is physical adsorption of the CNTs/GA on the surface. This finding come in agreement with the results achieved with E_a_ values. It was noticed that low and almost close values of E_a_ and ΔH_a_ were obtained showing that the increase of temperature maintained physical adsorption with no change in corrosion mechanism, which is here pitting attack [62,66,67].

As for the ΔS_a_ values, negative signs for all solutions signify the freely moving CNTs and/or GA molecules in the deionized water. GA-deionized water solutions had higher entropy values than the corresponding CNTs nanofluids as the latter solutions contain more species hence movements would be restricted [61].

### 3.4. Scanning Electrons Microscope (SEM) Observations

After testing the 316L stainless steel samples in 0.1 and 1.0 wt.% CNTs–water nanofluids with OCP (10 h), polarization resistance and then potentiodynamic tests, samples were removed from the solution and were left to air-dry before being observed with the SEM. A clear black layer of CNTs was observed to cover the surface of the samples tested in CNTs nanofluids and this layer seemed to be thicker for the samples tested in 1.0 wt.% CNTs nanofluids.

**Figure 7:**
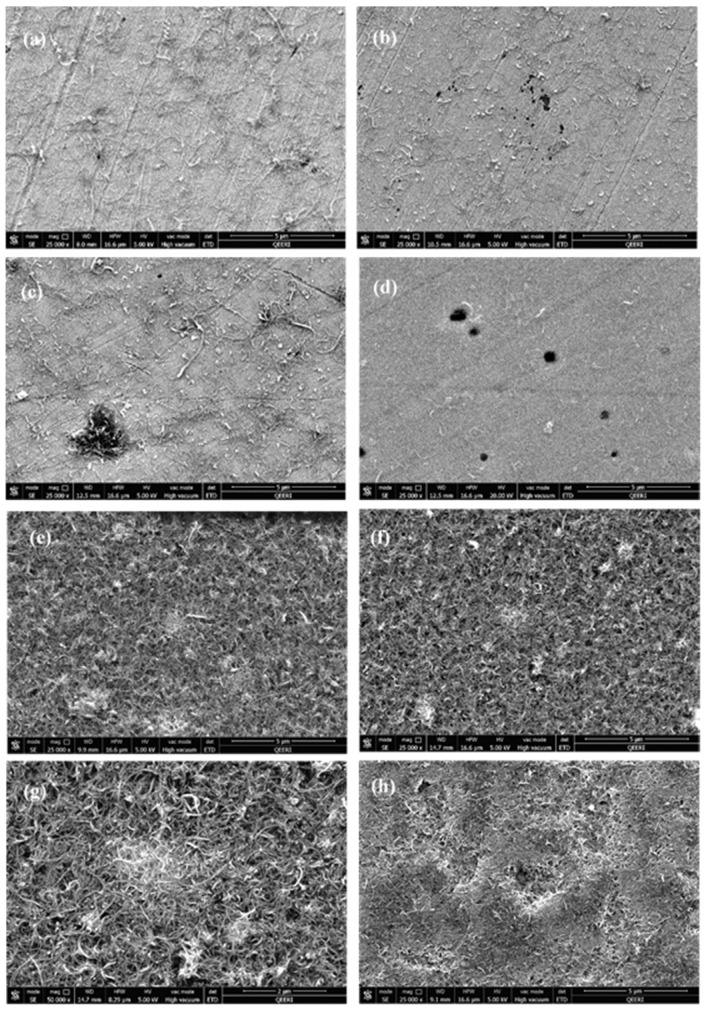
SEM images before washing for 316L stainless steel samples tested in (**a**–**d**) 0.1 wt.% CNT-water nanofluids at (**a**) 22 °C (**b**) 40 °C (**c**) 60 °C (**d**) 80 °C. Figure (**e**–**h**) tested in 1.0 wt.% CNTs–water nanofluids at (**e**) 22 °C (**f**) 40 °C (**g**) 60 °C (**h**) 80 °C.

Figure 7 shows the SEM images of the samples examined in both nanofluids at different temperatures before washing. In these Figures, tangled CNTs appeared with discrete white areas on their surfaces due to the presence of GA molecules. For the samples tested in 0.1 wt.% CNTs (Figure 7a–d), the distribution of CNTs appears to be random and covering parts of the surface, while some corrosion pits are present on the surface as well. On the other hand, CNTs distribution seemed to be more uniform and covering most of the surface of samples tested in 1.0 wt.% CNTs nanofluids (Figure 7e–h). However, some areas of the surface of the metal were exposed to test solution left uncovered and caused creation of empty spaces between unevenly accumulated CNTs, as it appears from some holes formed among gathered CNTs on the surface of the metal. After removing the black layer with deionized water, pits were visibly spotted on the surface.

**Figure 8:**
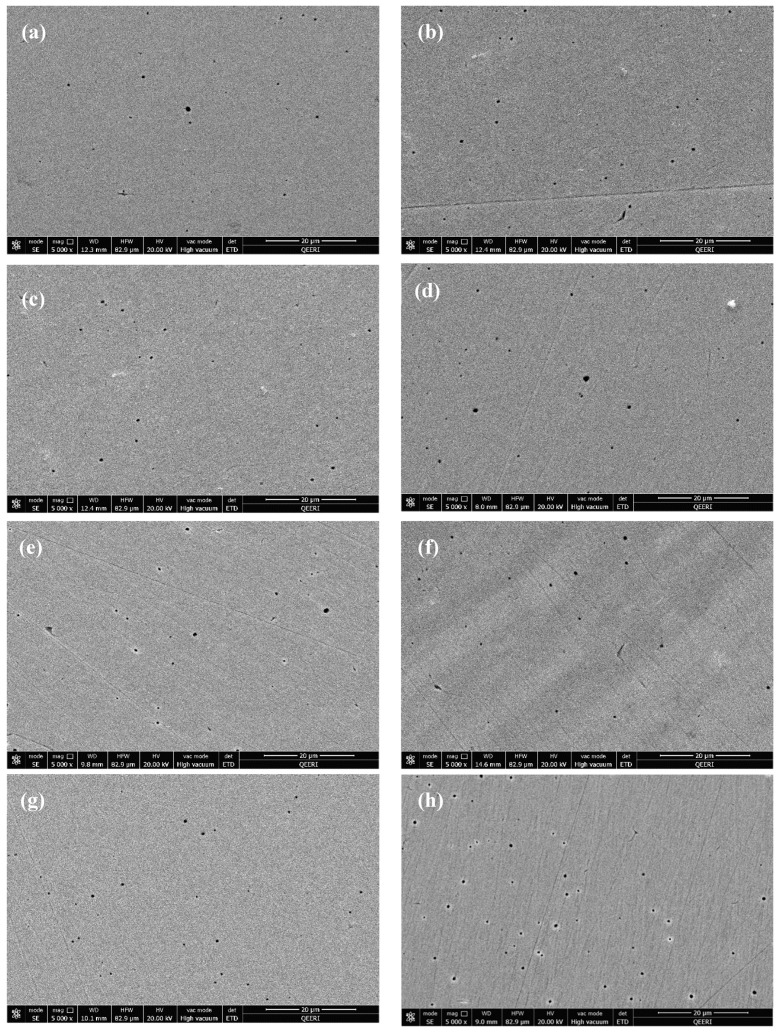
SEM images after washing for 316L stainless steel samples tested in (**a**–**d**) 0.1 wt.% CNT-water nanofluids at (**a**) 22 °C (**b**) 40 °C (**c**) 60 °C (**d**) 80 °C. Figures (**e**–**h**) tested in 1.0 wt.% CNTs–water nanofluids at (**e**) 22 °C (**f**) 40 °C (**g**) 60 °C (**h**) 80 °C.

After washing the samples with deionized water, pits diameter and density were inspected. As seen in Figure 8, pits density and diameter revealed to increase with increasing the corrosion rates of the tested samples. Samples tested in 0.1 wt.% CNTs nanofluids had almost same pits diameter but lower pits density with respect to the samples tested in 1.0 wt.% nanofluids at the corresponding temperatures. The examined pits of all samples were found to have diameter in the range of 0.25–1.15 µm.

### 3.5. Optical Profiler Observations

**Figure 9:**
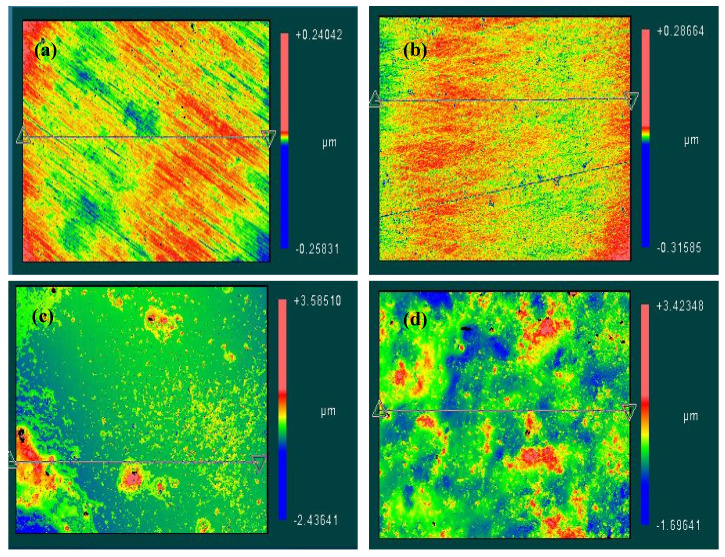
Optical surface profiler images for selected 316L stainless steel samples after corrosion testing in (**a**) 0.1 wt.% CNTs nanofluid at 60 °C (**b**) 0.1 wt.% CNTs nanofluid at 80 °C (**c**) 1.0 wt.% CNTs nanofluid at 60 °C (**d**) 1.0 wt.% CNTs nanofluid at 80 °C.

An optical profiler was also used to image the distribution of CNTs and to measure the superficial roughness of the 316L stainless steel surfaces tested in low and high CNTs loadings of 0.1 and 1.0 wt.% CNTs–water nanofluids, respectively. Selected profiler images are presented in Figure 9 that show the scattering of CNTs on the metal surface along with the superficial roughness due to the adsorption of CNTs/GA on the metallic surface. In general, the distribution and roughness of samples were mainly influenced by the exposed CNTs concentration regardless of tested temperature.

Figure 9a,b show profiler images of the steel after the corrosion experimentation in 0.1 wt.% CNTs–water nanofluid at 60 °C and 80 °C, respectively. These images showed the surface have some external fluctuations. On the other hand, the steel tested in 1.0 wt.% at the same temperatures (Figure 9c,d) had apparent CNTs accumulations distributed randomly within the surface that caused higher surface roughness compared to the samples of lower CNTs concentrations. Average surface roughness detected was in the range of (5–10) and (160–280 nm) for samples tested in 0.1 and 1.0 wt.% CNTs nanofluids, respectively.

### 3.6. Contact Angle Analysis

CNTs addition to the GA-deionized water solutions resulted in lower corrosion rates of the 316L stainless steel samples as it was indicated by the increase in the inhibition efficiency values. Pristine CNTs have hydrophobic characteristics in nature [68], and it has been identified that hydrophobic surfaces can retard electrochemical reactions [69]. To achieve corrosion reduction on metal surfaces, superhydrophobic coatings are fabricated to have a suitable surface roughness with low surface energy materials [70]. The presence of such conditions allow air molecules to entrap within the micro/nano structure of the rough surface, which will prevent corrosive ions from attacking the surface [71].

In a way to understand the inhibition mechanism of CNTs, the contact angle analysis was performed to examine whether the hydrophobicity of CNTs had an influence in decreasing corrosion of the surface of the steel. Therefore, tested stainless steel samples with the CNTs layer (unwashed) were evaluated using contact angle and values of the contact angle were reported in Table 7. The surface of all samples, including that for clean metal without the CNTs layer, are revealed to be hydrophilic since the values of the contact angle were less than 90° [72]. This was due to the attachments of GA molecules to the hydrophobic surface of CNTs. GA molecules in contact with the CNTs changed the surface properties of the CNTs in order to allow their dispersion in the deionized water. Hence, hydrophobic nature of the CNTs did not contribute to the inhibition corrosion of the steel as the presence of the GA surfactant changed the hydrophobic characteristics of the CNTs surface to hydrophilic.

## 4. Conclusions

The presence of CNTs and GA in CNTs–water nanofluid was shown to influence the corrosion behaviour of the 316L stainless steel, especially at higher temperatures. As indicated from the potentiodynamic scans, passivity behaviour (oxides formation) on the surface of the metal changed at higher temperatures (60 and 80 °C) for both CNTs concentration. Pitting potential values were lower for higher concentrations and at higher temperatures, indicating a greater possibility of pit initiation at these conditions. In addition, the presence of these species has also affected both anodic and cathodic reactions, as noted from the change in the corrosion potential values (E_corr_). Their influence was higher on anodic reactions as they worked on retarding the corrosion rate by occupying active anodic sites, which is considered part of the blocking geometry effect.

CNTs contributed in corrosion inhibition of the stainless steel, but this inhibition decreased with temperature. Compared to GA-deionized water solutions, CNTs added extra resistance to the solution and decreased the corrosion rate at all temperatures. The lowest corrosion rate obtained was 6.43 milli-mpy (milli-mil per year) for the steel tested in 0.1 wt.% CNTs nanofluids at 22 °C, while the highest corrosion rate had a value of 32.66 milli-mpy that was obtained in the 1.0 wt.% CNTs nanofluid at 80 °C. CNTs and GA species formed a thin layer that is electrostatically adsorbed to the surface of the metal. Such physical and reversible adsorption of these species caused them to desorb at higher temperatures. Another factor that affected the corrosion rate is the non-homogenously distributed CNTs on the surface of the metal. The presence of CNTs was shown to increase the roughness of the surface especially for the 1.0 wt.% CNTs nanofluid. Such unevenly accumulated CNTs can form active anodic sites beneath them. Finally, the hydrophobicity of CNTs did not impact the corrosion behaviour, as the surface properties of the CNTs have changed due to the attachments of the GA species to their surfaces.

## Figures and Tables

**Table 1:** Specifications of MWCNT used in the present research.

Outer Diameter (nm)	Inside Diameter (nm)	Purity (%)	Length (µm)	Specific Surface Area (m^2^/g)	Electrical Conductivity (S/cm)	Bulk Density (g/cm^3^)	True Density (g/cm^3^)
20–30	3–10	>95	10–30	110	>100	0.28	~1.2

**Table 2:** Composition of tap water used in testing [40].

Constituent	Bromide (Br^−^)	Bicarbonate (HCO_3_^−^)	Carbonate (CO_3_^−2^)	Fluoride (F^−^)	Chloride (Cl^−^)	Nitrate (NO_3_^−^)
Amount (mg/Liter)	0.3	76.9	<1.0	0.1	16.3	<0.01
Constituent	Total Organic Carbon (TOC)	Total Nitrogen	Sulphate (SO_4_^−2^)	Calcium (Ca)	Magnesium (Mg)	Total Dissolved Solids (TDS)
Amount (mg/Liter)	0.3	0	<2.0	25	1.2	92

**Table 3:** Composition of 316L stainless steel (weight percentages) purchased from the company Metals Samples A Division of Alabama Specialty Products, Inc.

Fe	Cr	Ni	Mo	C	Mn	Si	Cu	Co	Others
68.28	16.65	10.10	2.03	0.02	1.48	0.48	0.46	0.37	0.13

**Table 4:** Corrosion potential values for 316 L stainless steel tested in CNTs nanofluids and GA solutions at different temperatures.

Solution (Compositions in wt.%)	Temp. (°C)	Ecorr (mV_SCE_)	ΔEcorr (mV_SCE_)
0.1% CNTs + 0.3% GA	22	−38	83
	40	−48	42
	60	−54	6
	80	−70	6
0.3% GA	22	−121	-
	40	−90	-
	60	−60	-
	80	−77	-
1.0% CNTs + 3.0% GA	22	−28	66
	40	−46	61
	60	−61	10
	80	−53	29
3.0% GA	22	−94	-
	40	−107	-
	60	−71	-
	80	−82	-

**Table 5:** Polarization parameters for 316 L stainless steel tested in CNTs nanofluids and GA solutions at different temperatures.

Solution (Compositions in wt.%)	Temp. (°C)	Βa (V/Decade)	Βc (V/Decade)	Epit (V_SCE_)	Icorr (µA/cm^2^)	Rp (Kohm)	Corrosion Rate (Milli-mpy)	IE (%)
0.1 % CNTs + 0.3% GA	22	0.2644	0.1384	1.13	0.020	5950	6.43	57.9
	40	0.4007	0.1001	0.95	0.043	2688	11.30	37.5
	60	0.3244	0.1889	0.76	0.062	2894	17.11	29.1
	80	0.3202	0.1143	0.64	0.087	1231	27.03	24.9
0.3% GA	22	0.4726	0.1533	1.05	0.049	3091	15.25	-
	40	0.3569	0.1496	0.93	0.058	2464	18.07	-
	60	0.1144	0.0754	0.82	0.078	1645	24.13	-
	80	0.1243	0.0808	0.80	0.118	562	36.00	-
0.1 % CNTs + 0.3% GA	22	0.2564	0.1367	0.99	0.039	3028	12.06	9.0
	40	0.3266	0.1900	0.91	0.058	2700	17.95	8.7
	60	0.3403	0.2136	0.85	0.072	2382	22.43	7.0
	80	0.2534	0.1076	0.75	0.109	885	32.66	3.5
3.0% GA	22	0.3030	0.1182	1.01	0.043	2615	13.25	-
	40	0.7562	0.2066	0.95	0.063	3445	19.66	-
	60	0.8833	0.1747	0.87	0.078	2402	24.12	-
	80	0.4579	0.1337	0.76	0.105	1290	33.86	-

**Table 6:** Calculated values of thermodynamic parameters for 316L stainless steel tested in 0.1 and 1.0 wt.% CNTs–water nanofluids.

Solution	Ea (kJ/mol)	ΔHa (kJ/mol)	ΔSa (J/mol/K)
0.1 wt.% CNT nanofluid	21.1	18.433	−256.5
1.0 wt.% CNT nanofluid	14.3	11.648	−317.4
0.3 wt.% GA solution	12.7	10.046	−335.0
3.0 wt.% GA solution	13.5	10.811	−329.7

**Table 7:** Contact angle values for the surface of 316L stainless steel samples after corrosion testing in 0.1 and 1.0 wt.% CNTs nanofluids at different temperatures.

Solution	Temp. (°C)	Contact Angle (°)
Clean surface	22	59.1
0.1 wt.% CNTs–water nanofluid	22	64.1
	40	57.0
	60	52.6
	80	50.4
1.0 wt.% CNTs–water nanofluid	22	51.0
	40	63.5
	60	79.3
	80	83.4

## Data Availability

The data presented in this study are openly available.

## Nomenclature

GA	Gum Arabic
CNTs	Carbon nanotubes
HTF	Heat transfer fluid
MWCNTs	Multi-walled carbon nanotubes
SCE	Saturated calomel electrode
OCP	Open circuit potential
SDS	Sodium dodecyl sulfonate
SDBS	Sodium dodecyl benzene sulfonate
